# Sub-acute hypersensitive reaction to botulinum toxin type A following Covid-19 vaccination: Case report and literature review

**DOI:** 10.1097/MD.0000000000027787

**Published:** 2021-12-10

**Authors:** Xiaoshuang Guo, Tongtong Li, Ye Wang, Xiaolei Jin

**Affiliations:** Chinese Academy of Medical Sciences & Peking Union Medical College, Plastic Surgery Hospital, China.

**Keywords:** allergy, botulinum toxin type A, case report, Covid-19, vaccine

## Abstract

**Rationale:**

Botulinum toxin type A (BTA) is one of the most widely used injectable agents in cosmetic surgery. Corona virus disease 2019 (Covid-19) infection and vaccination, which can induce specific and nonspecific activation of the immune system, has been reported to induce delayed inflammatory reactions to previously injected hyaluronic acid fillers. However, there are no reports about the interaction between BTA and Covid-19. We aimed to report 2 sub-acute cases of allergic reactions to BTA in facial cosmesis following the Covid-19 vaccination.

**Patient concern:**

A 35-year-old and a 34-year-old female who has several previous BTA injections without any adverse effects experienced facial swelling, flu-like symptoms after BTA treatment following the Covid-19 vaccination.

**Diagnose:**

According to the typical clinical manifestation, a hypersensitive reaction to BTA was considered.

**Intervention:**

Corticosteroids and antihistamine were administered empirically.

**Outcomes:**

The flu-like symptoms recovered over the next day, but the facial swelling gradually faded within 1 to 2 weeks.

**Lessons:**

A literature review was also conducted to summarize the hypersensitive actions to cosmesis related to Covid-19. We recommend BTA injection be administered at least 2 to 3 months after Covid-19 vaccination.

## 1 Introduction

Botulinum toxin type A (BTA) is the purified neurotoxin produced by *Clostridium botulinum*. By reversibly inhibiting neurotransmitter release, BTA induces flaccid muscle paralysis and exhibits reliable efficacy in reducing muscular activity and tonicity.^[1]^ Nowadays, BTA is the most widely used injectable agent both for cosmetic and therapeutic purposes. Its in-label and off-label use are constantly expanding due to its efficacy and rare reports of severe side effects.^[2]^ Meanwhile, an increasing number of adverse effects was also reported according to the FDA Adverse Event Reporting System.^[3]^

The Corona virus disease 2019 (Covid-19) epidemic has wreaked havoc on the world by altering many facets of our lives. Nowadays, there are more than 181 million coronavirus cases, and the world has stepped into an era of vaccination. Although truly life-threatening complications related to Covid-19 are scarce for cosmetic purposes, practitioners should still be well-versed in the clinical manifestations and treatment of possible rare adverse events.

Herein, we reported 2 sub-acute cases of hypersensitive reactions to Chinese BTA (named Prosigne in Brazil, Lanzhou Institute of Biological Products, China) in facial cosmesis following Covid-19 vaccination.

## 2 Case presentation

### 2.1 Patient 1

**Figure 1:**
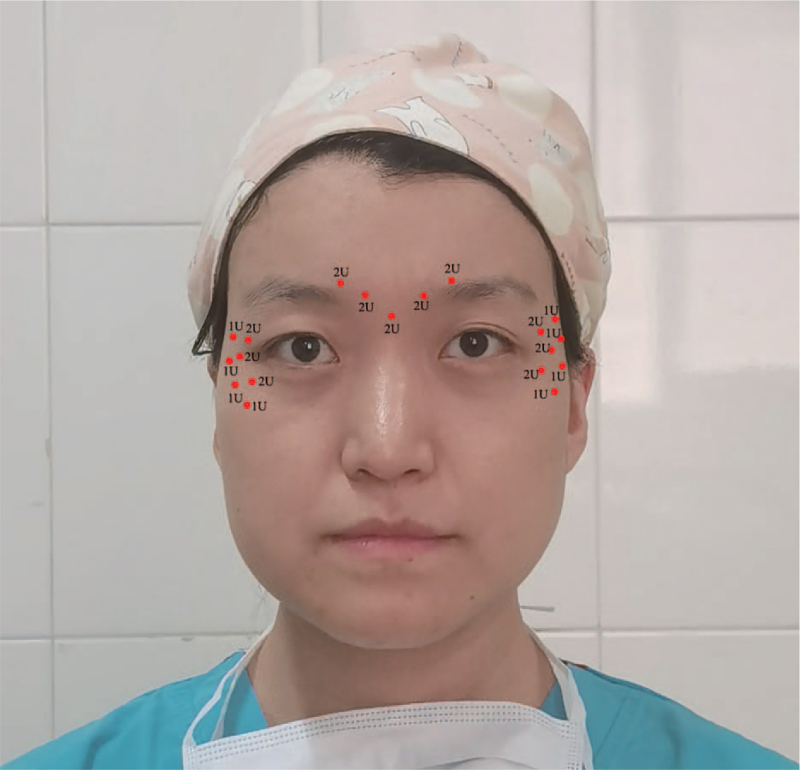
Pre-injection photographs of case 1. The red dots illustrated the injection site and dosage.

A healthy 35-year-old female anesthetist experienced facial swelling, flu-like symptoms, and headache after the Chinese BTA injection in the glabella and crow's feet. She had BTA injections 4 times previously for the treatment of masseter hypertrophy without adverse events. The first 2 injections were Chinese BTA, and the last 2 shots were Botox (30-50U on each side). She was inoculated against COVID-19 using SARS-Cov-2 Vaccine (Vero Cell, inactivated vaccine, Sinovac life sciences Co., LTD, Beijing, China) 2 months before BTA injection and had a booster dose after 2 weeks. Furthermore, she reported a history of mild allergic rhinitis in the recent month without any medications. There were no significant findings in her family history (Fig. 1).

**Figure 2:**
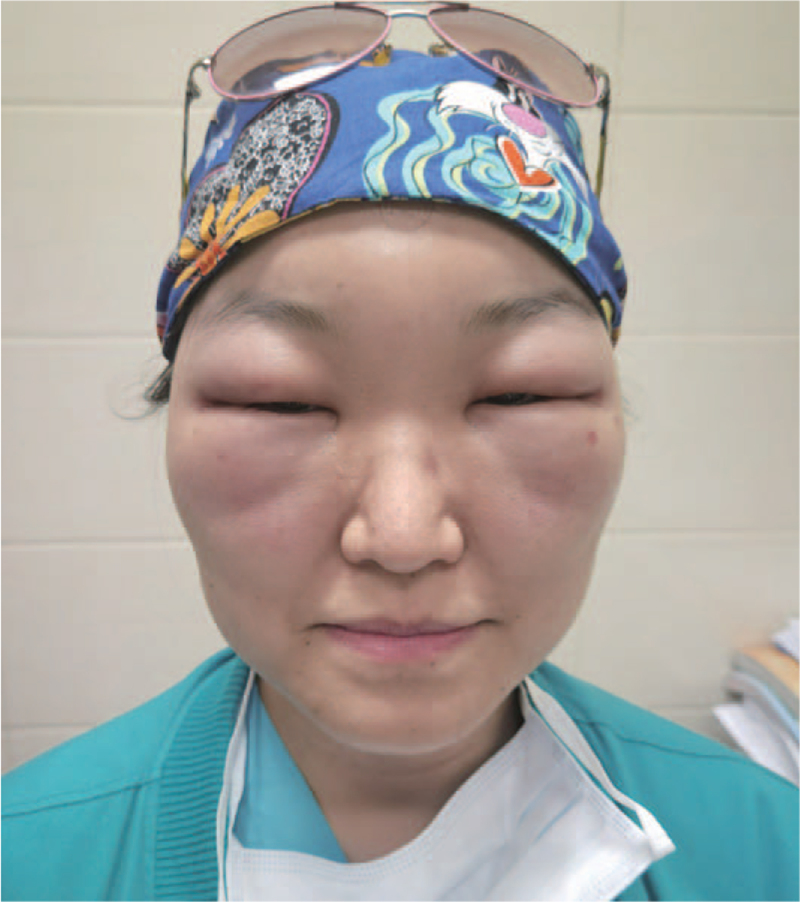
Eleven hours’ photographs after BTA injection of case 1. Note the whole face weals gradually following botulinum toxin type A injection into the glabella and crow's feet. Distinct epiphora can also be seen from the pictures. Her vision is largely limited due to the swelled eyelids. Then dexamethasone 10 mg, calcium gluconate 10 mg, and Loratadine tablets 10 mg are administered immediately muscles.

She received 10 U of Chinese BTA in the glabella and 20 U in the crow's feet (10U on each side). She was not in the menstrual period. Before injection, no local anesthetics were used, and the BTA compound was prepared by mixing 2.5 ml of 0.9% sodium chloride and 100 U Chinese BTA (Lanzhou Institute of Biological Products, China; Production No. 20201087; Date of manufacture: October 26, 2020; Date of expiration: October 25, 2023). She was allowed to discharge after 20 minutes’ observation. About 3 hours later, she experienced a gradual swelling in the periocular region. As a doctor herself, 10 mg of Loratadine tablets (Clarityne, Bayer, Germany) were taken. Around 11 hours later, she had flu-like symptoms with constant epiphora and rhinorrhea, mild headache, swollen face, limited vision due to swollen eyelids. Then, she rushed to the emergency department (Fig. 2).

**Figure 3:**
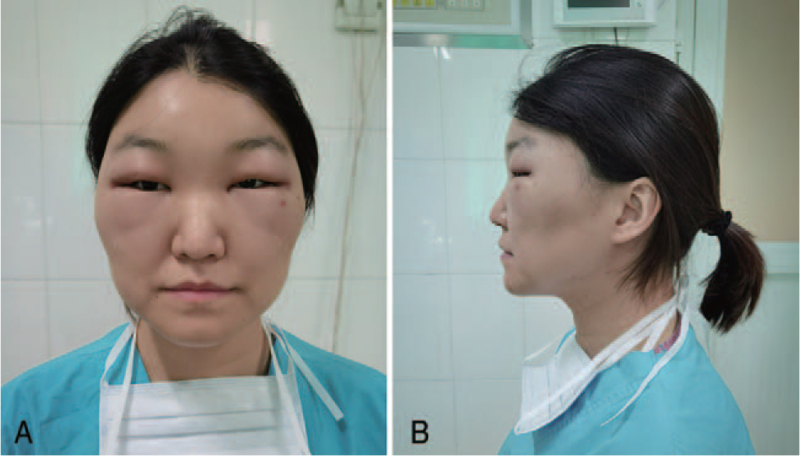
Fifteen hours’ photographs after BTA injection of case 1. Three hours after the use of glucocorticoid and antihistamine, the swollen face subsides. (A) Frontal view. (B) Lateral view.

**Figure 4:**
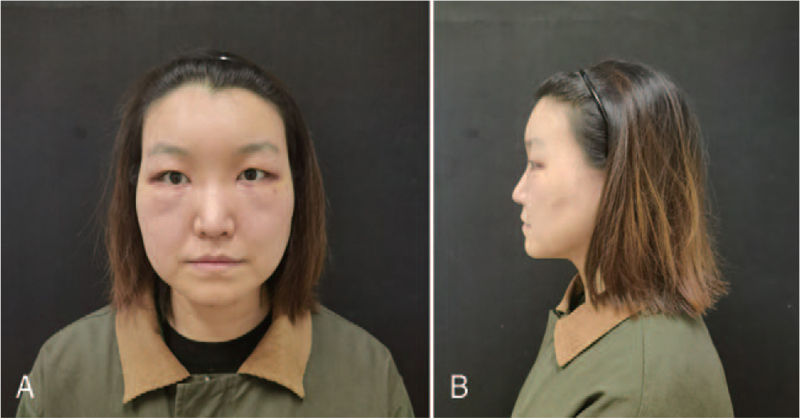
Three days’ photographs after BTA injection of case 1. Note the bruise of the needle puncture in the left crow's feet. (A) Frontal view. (B) Lateral view.

**Figure 5:**
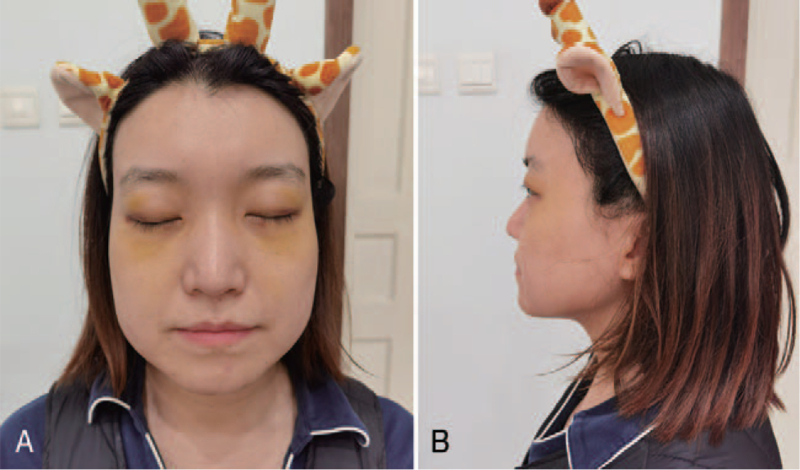
Seven days’ photographs after BTA injection of case 1. After 3 d treatment with glucocorticoid and antihistamine, the patient's face is fully recovered. (A) Frontal view. (B) Lateral view.

On examination, the patient showed a blood pressure of 117/70 mm Hg, a pulse of 120 bpm, a respiratory rate of 18, oxygen saturation of 98% breathing room air. The breath south was clear without rales. Dexamethasone 10 mg, calcium gluconate 10 mg, and Loratadine tablets 10 mg were administered immediately. The patient was referred to the observation unit for 24 hours observation in case of dyspnea or dysphagia. The symptoms gradually faded away over the next day. The same regime and intermittent ice pack for early detumescence were used for 3 consecutive days. All symptoms recovered after 7 days, and the patient received satisfactory result with less noticeable wrinkles. (Figs. 3-5)

### 2.2 Patient 2

A 34-year-old female experienced generalized facial swelling and flu-like symptoms after a Chinese BTA injection in the glabella. She was inoculated against COVID-19 using SARS-Cov-2 Vaccine (Vero Cell, inactivated vaccine, Sinovac life sciences Co., LTD, Beijing, China) 2 weeks before the BTA injection. The patient's family history was unremarkable, and she had BTA injection 2 times previously without any adverse events.

She was not in the menstrual period. Before injection, no local anesthetics were used, and 100 U Chinese BTA (Lanzhou Institute of Biological Products, China) was diluted into 2.5 ml of 0.9% sodium chloride. After 20 minutes’ observation, she was allowed to go home. However, within a few hours, she started to experience swollen lower face and flu-like symptoms. The symptoms gradually aggravated over the injection night. No respiratory distress or dysphagia was reported. The following morning, the patient went to an emergency room, where she was given dexamethasone and antihistamine. The dyspnea gradually alleviated over the next day, but the swelling receded slowly in the following 2 weeks. And the patient was dissatisfied with the treatment due to this adverse event.

## 3 Materials and methods

**Figure 6:**
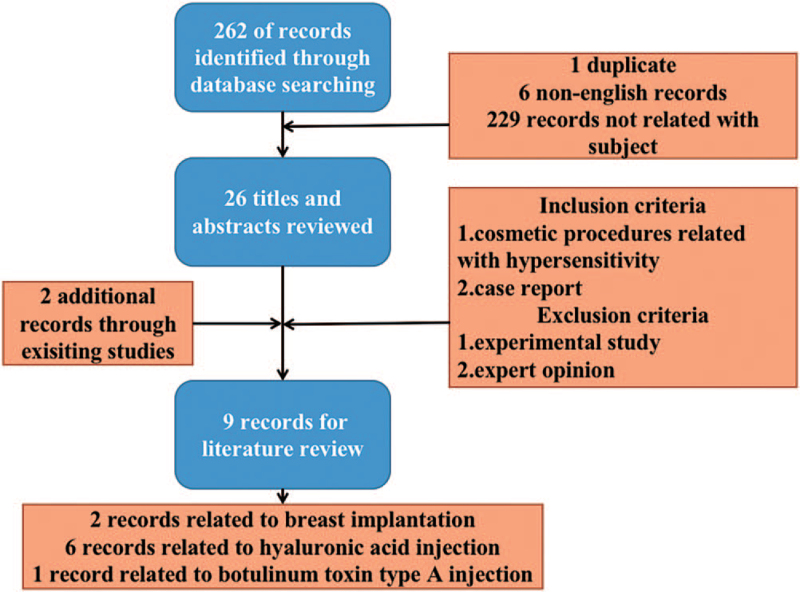
Flowchart of the literature screening.

A systematic search for all relevant articles up to October 2021 was conducted in Pubmed. The search strategy combined following Medical Subject Headings and keywords (Covid-19, cosmetic injection, botulinum toxin, hyaluronic acid, plastic surgery, cosmesis). Reference lists of all eligible studies and relevant reviews were manually searched for any additional reports. We only included English-language articles, though articles in other languages were also summarized (Fig. 6, Table 1).

**Table 1:** Summary of the previous published articles concerning allergic reaction to BTA in cosmetic treatment.

Study	Country	Previous history concerning BTA	Brand/dosage of BTA	Co-administration of medications	Symptoms	Treatment	Mechanism	Begin and duration of the symptoms
Rosenfield et al^[11]^	USA	Three times of Botox injection	Botox; 20U in the forehead and 12 U in the glabella;	Juvederm	Generalized itchness; small flat red dot at the injection site	Methylprednisolone for 5 d	Type IV, T-cell-mediated allergy	36 h;3 wks
Careta et al^[10]^	Brazil	No BTA injection before	Prosigne; total 32U in glabella, orbicularis, frontalis	No	Urticarial plaques proximal to the injection site	Corticosteroids/antihistamine	Allergic reaction	20 min;72 h
Moon et al^[8]^	Korea	One time of Botox injection; allergic rhinitis	Vistabel; 50U in the masseter muscle (25U in each side)	EMLA cream (containing lidocaine/prilocaine)	Severe rhinorrhya/nasal obstruction/swollen eyes/weals on extremities/mild dyspnoea/chest tightness	Epinephrine 1mg/chlorpheniramine 4 mg/diphenylpyraline 3 mg/dexamethasone disodium phosphate 5 mg	Type I, IgE	5 mins; The nasal obstruction and rhinorrhea alleviated 1 h after treatment and periocular swollen lasted for 4 h
Mya et al^[7]^	Malaysia	Five times of BTA injection	NA; temporal area	NA	Expired suddenly	Hydrocortisone 200 mg/adrenaline 500 mg	Anaphylaxis	Collapsed soon after injection

## 4 Discussion

BTA is one of the most commonly used cosmetic injectant. Since the first approval in 1989, there has been a tremendous increase in its usage and range of use. In 2020, more than 4 million cosmetic BTA procedures were performed in the USA alone, exhibiting a 459% increase compared with 2000.^[4]^ Furthermore, due to the predictable adverse events and favorable efficacy, we witnessed a tremendous expansion of its off-label treatment for cosmetic, neuromuscular, and dermatic conditions.^[5]^ With increasingly convenient access to Covid-19 vaccination, even in the Covid-19 epidemic, millions of BTA injections are still adminstered worldwide. A rising number of reported adverse events coupled with the popularity of BTA treatment, which indicates the importance of gaining appropriate knowledge about its complications and long-term safety.^[6]^

Herein, we reported 2 cases of sub-acute hypersensitive reactions to BTA following covid-19 vaccination, which might develop into life-threatening symptoms. Two particular concerns of these cases were: previous Covid-19 vaccination; and the sub-acute, progressive manifestation of allergic reaction that was different from previously reported anaphylaxis.^[7–9]^

The present report is the first subacute hypersensitive reaction to BTA following inactivated virus Covid-19 vaccination. We postulated 2 possible mechanisms, of which 1 was the patients be allergic to BTA or bovine gelatin, an excipient in Chinese BTA, the other was the hypersensitive status to injectant following Covid-19 vaccination.

Regarding allergic reaction to BTA in cosmesis, a total of 4 cases has been reported so far. Among them, 2 patients had localized symptoms, and 2 patients had generalized symptoms.^[7,8,10,11]^ Two patients were allergic to Botox/Vistabel, and 1 patient showed allergy to Prosigne, while 1 patient died from an unspecified BTA. Only 2 cases further explored the mechanism of allergy by prick/intradermal/patch test, and found Gell-Coombs type IV, T-cell-mediated hypersensitivity allergy or type I, IgE-mediated anaphylaxis. Given that the previous sequential BTA treatments were uneventful, the possibility of the 2 patients in the present study both being caused by allergy is not large. Besides, the allergy was unlikely to be caused by neurotoxin antibody stimulated through repeated BTA treatment because the wrinkles were less noticeable 7 days after the injection. Additionally, both patients recovered speedily after corticosteroid and histamine treatment. Thus, it is less likely to be caused by local infection.

The World Health Organization had mainly approved 3 kinds of Covid-19 Vaccines, including mRNA vaccine (Pfizer, Moderna), viral vector vaccine (AstraZeneca, Sputnik V), and inactivated virus vaccine (CoronaVac, Sinopharm).^[12]^ For an efficient vaccination, an antibody or T-cell mediated response should be evoked, and this elicited immune status may cause hypersensitivity in cosmesis. A literature review of Pubmed was performed, and the summarized report was listed in Table 2. Restifo^[13]^ reported 1 case of capsular contracture at 6 months after mammaplasty with implants following Covid-19 vaccination. Left breast firmness with enlarged lymph node appeared after 2 doses of Pfizer vaccines both placed in the ipsilateral shoulder. The symptoms rapidly progressed to Baker IV contracture, and revision surgery was administered. Weitgasser et al^[14]^ further introduced 4 cases of potential reactions associated with breast implants following the Covid-19 vaccination. Meanwhile, reactions to dermal filler after vaccination were also reported. Zhang et al^[15]^ depicted facial or lip swelling in 3 patients who had previous soft filler injections after the Moderna Covid-19 vaccination. Shome^[16]^ and Rowland-Warmann et al^[17]^ described 2 patients with hypersensitive reactions to hyaluronic acid after Covid-19 infection. Meanwhile, Munavalli et al,^[18]^ Michon,^[19]^ and Savva et al^[20]^ further detailed 6 additional cases of inflammatory reaction to hyaluronic acid composed of 5 cases after Covid-19 mRNA vaccination and 1 case after Covid-19 infection. Akdogan et al^[21]^ reported 1 case of severe hyperalgesia during BTA injection after Covid-19 infection. In summary, hypersensitivity to BTA following Covid-19 vaccination may be the possible mechanism of the present 2 cases.

**Table 2:** Summary of the previous published articles concerning hypersensitive reaction in cosmesis related to Covid-19.

Study	Country	Covid-19 related medical history	Symptoms	Treatment	Previous cosmetic procedure
Restifo^[13]^	USA	Pfizer vaccine	Enlarged lymph node in the left axilla at 6 d after the 2nd dose. Left breast firmness, swelling, tightness at 13 d after the 2nd dose. The breast progressed to Baker IV capsular contracture.	Oral intake of Monelukast, capsulectomy and implant exchange	Mammaplasty with implants 6 mo previously.
Weitgasser et al^[14]^	Germany	One shot of Biotec vaccine	Bilateral breast pain and swelling 2 d after the 1st dose.	Oral NSAIDS/cryotherapy	Mammaplasty with implants 5 yrs previously.
		One shot of Biotec vaccine	Bilateral breast pain and redness 2 d after the 1st dose.	Oral NSAIDS/cryotherapy/antibiotic	Mammaplasty with implants 17 mo previously.
		One shot of Johnson & Johnson vaccine	Unilateral breast pain 3 d after the 1st dose.	Oral opioid and metamizole	Mastectomy and expander implantation 9 mo previously.
		One shot of AstraZeneca vaccine	Unilateral pain, inflammation, and seroma 1 d after the 1st dose.	Implant removal and autologous breast reconstruction, parenteral antibiotics	Mastectomy and implant reconstruction 2 mo previously.
Zhang^[15]^	USA	Moderna vaccine phase-3 trail	Facial swelling 1 d after the 2nd dose.	Antihistamine or steroid course	Hyaluronic acid injection in the face 6 mo previously.
		Moderna vaccine phase-3 trail	Facial swelling 2 d after the 2nd dose.		Hyaluronic acid injection in the face 2 wks previously.
		Moderna vaccine phase-3 trail	Lip angioedema 2 d after vaccination.		Previous hyaluronic acid injection (time interval not available).
Shome et al^[16]^	India	Covid-19 infection	Sudden swelling in the periocular area at 1 mo after tested positive for Covid-19.	Oral anti-inflammatory treatment	Hyaluronic acid injection in the face 10 mo previously.
Rowland-Warmann^[17]^	UK	Covid-19 infection	Edema, induration, erythema, mild tenderness, tightness around the radix at 3 wks after tested positive for Covid-19.	No medical intervention	Hyaluronic acid injection in the nose 5 mo previously.
Munavalli^[18]^	USA	Covid-19 infection	Swelling and burning feeling in the lip, cheek and tear trough 2 wks after tested positive for Covid-19.	Steroid/hyluronidis/antibiotic/microneeding	Hyaluronic acid injection in the nose 1 mo previously.
		Moderna Covid-19 vaccine	Tenderness and edema in the perioral edema at the day she received the first dose.	Antihistamine/lisinopril	Hyaluronic acid injection in the nose 1 yr and a half previously.
		Pfizer vaccine	Mild tenderness and swelling in the left eye 1 d after the second dose.	Steroid	Hyaluronic acid injection in the nose 2 yr and a half previously.
Michon^[19]^	Canada	Covid-19 vaccination	Two patients had delayed reactions after hyaluronic acid treatment of the tear trough (details not available).	No medical intervention for the first patient and hyaluronidase injection for the other one	Hyaluronic acid injection in the face months previously.
Savva et al^[20]^	Italy	Pfizer vaccine	Erythematous nodules, tenderness, and pain of the lip.	Oral methylprednisolone	No previous hyaluronic acid injection.
Akdogan^[21]^	Turkey	Covid-19 infection	One patient experienced a VAS 10-point pain. In the previous injection, the pain was rated 3.	Injection stopped	BTX injection for facial wrinkles every 4-6 mo for 7 yrs.

Of the millions of cosmetic BTA procedures administered annually, most adverse events are transient and self-limited. Generalized complications such as dysphagia, flu-like symptoms, allergy are infrequently reported. However, due to the worldwide underreporting, complications may be overlooked. One to three rare adverse events gathered from the spontaneous reporting system are not likely to be a coincidence.^[21]^ With the increasingly convenient access to Covid-19 vaccine and the popularity of BTA treatment, a few occurrences now might indicate a phenomenon that could become more prevalent in the general population and should not be overlooked.

Another special point of the present case series is the subacute process. According to a previous report, the onset of anaphylaxis is usually rapid, with 70% of the cases begin within 20 minutes. Although the late-phase reaction can occur several hours after the early reaction, it seldomly happens without primary hypotension or airway distress.^[22]^ Both of the patients experienced gradually swollen faces and flu-like symptoms overnight. The danger was that the onset of symptoms was few hours after the injection, which exceeded the required observation period in the clinic.

This report indicates that subacute hypersensitive reactions to BTA may happen following the Covid-19 vaccination. Although the exact treatment intermission is not defined, at least 2 to 3 months interval is suggested. Furthermore, thorough pre-injection information of vaccine acquisition, early awareness of possible symptoms, and timely treatment are critical to avoid exacerbating the sub-acute reactions into life-threatening side effects. Last but not least, the vaccine's benefits exceed its possible dangers, and vaccination should be encouraged for the patient's sake.

The limitation of this report is that both patients refused a timely blood test because their symptoms recovered promptly. Thus, only empirically differential diagnosis was proposed.

## 5 Conclusion

These case reports serve as a cautionary note for plastic surgeons that life-threatening adverse events after cosmetic BTA treatment may happen following Covid-19 vaccination. However, vaccination should still be encouraged for the patient's sake.

## Acknowledgments

We thank patient No.1 for providing her photographs, and thank patient No.2 for sharing her medical records.

## Author contributions

**Conceptualization:** Xiaoshuang Guo.

**Data curation:** Xiaoshuang Guo, Tongtong Li.

**Formal analysis:** Xiaoshuang Guo, Ye Wang.

**Funding acquisition:** Xiaolei Jin.

**Supervision:** Ye Wang, Xiaolei Jin.

**Writing – original draft:** Xiaoshuang Guo.

**Writing – review & editing:** Ye Wang, Xiaolei Jin.
